# Nephrectomy Does not Exacerbate Cancellous Bone loss in Thalassemic Mice

**DOI:** 10.1038/s41598-020-64681-2

**Published:** 2020-05-08

**Authors:** Sutada Lotinun, Korakot Atjanasuppat, Jutatip Limsuvech, Asada Leelahavanichkul, Saovaros Svasti, Nateetip Krishnamra

**Affiliations:** 1 Department of Physiology, Faculty of Dentistry, Chulalongkorn University, Bangkok, Thailand; 2 Skeletal Disorders Research Unit, Faculty of Dentistry, Chulalongkorn University, Bangkok, Thailand; 3 Department of Microbiology, Faculty of Medicine, Chulalongkorn University, Bangkok, Thailand; 4 Thalassemia Research Center, Institute of Molecular Biosciences, and Department of Biochemistry, Faculty of Science, Mahidol University, Bangkok, Thailand; 5 Department of Physiology, Faculty of Science, Mahidol University, Bangkok, Thailand

**Keywords:** Bone, End-stage renal disease

## Abstract

Patients with β-thalassemia have an increased risk of developing chronic kidney disease which is associated with osteoporosis and periodontitis. The purpose of this study was to evaluate mandibular and femoral bone change in heterozygous β-globin knockout (BKO) mice following 5/6 nephrectomy (Nx). Female and male BKO mouse blood smears demonstrated microcytic hypochromic anemia. Serum urea nitrogen, creatinine, calcium, and phosphorus levels were not changed in BKO mice. Nx increased the serum levels of urea nitrogen in both wild type (WT) and BKO mice and the level was much higher in BKO males. Serum level of creatinine was increased in Nx WT but not BKO mice. However, serum calcium and phosphorus levels were not altered. Nx induced comparable renal fibrosis in BKO mice and WT controls. Bone loss was observed in mandibular cancellous bone but not cortical bone of both male and female BKO mice. Nx decreased cancellous bone volume and cortical thickness in WT. Interestingly, BKO mice were resistant to Nx-induced cancellous bone loss. However, cortical thickness and cortical bone mineral density were reduced in Nx male BKO mice. Nx increased mRNA levels of *type I collagen*, *Osx* and *Trap* in WT but not BKO mice. Similarly, Nx reduced cancellous bone volume in femurs and increased osteoblast number and osteoclast number in WT not BKO mice. Serum FGF23 and erythropoietin levels were markedly increased in BKO mice. Nx decreased serum erythropoietin but not FGF23 levels. Since WT treated with erythropoietin exhibited a significant reduction in cancellous bone volume, it was possible that lower level of erythropoietin in Nx BKO mice prevented the Nx-induced cancellous bone loss.

## Introduction

β-thalassemia is an inherited disorder of hemoglobin synthesis in which point mutations of the β-globin gene cause defective β-chain production leading to an imbalance in α- and β-globin chain synthesis. β-thalassemia is characterized by ineffective erythropoiesis, hemolysis, splenomegaly, iron overload, anemia, growth retardation, frontal bossing with the early signs of abnormal thalassemic facies and skeletal deformity^1^. Three main forms of β-thalassemia, thalassemia major, thalassemia intermedia, and thalassemia minor, are classified according to severity of the disease^1^. Patients with β-thalassemia major present in the first year of life with profound anemia and subsequently require regular blood transfusions and iron chelation therapy for survival and end-organ damage prevention. However, some patients with β-thalassemia intermedia (hemoglobin level 7–10 g/dl) present later in life with a milder form of anemia and remain largely transfusion-independent. β-thalassemia minor patients or carriers are generally asymptomatic. In β-thalassemia patients, chronic anemia, iron overload from long-term blood transfusions, and specific iron chelation therapy are associated with renal impairment.

In the past, renal disease was not a major issue in thalassemia patients because they did not live long enough to develop conditions linked to renal dysfunction. Premature early death was apparently caused by severe cardiac iron loading from chronic transfusion therapy^2,3^. Recently, the use of effective chelating agents that can reduce the iron burden and its consequences helps to extend patients’ survival. Therefore, renal disease becomes a more common occurrence. Approximately 50% of transfusion-independent thalassemia intermedia patients developed glomerular hyperfiltration early in the course of the disease, and 14% of these patients developed proteinuria^4^. Long-term follow-up of these patients showed that 4.7% of thalassemia intermedia patients eventually developed end stage renal disease.

End stage renal disease or renal failure is the most advanced stage of chronic kidney disease (CKD) and is life-threatening. Patients require either kidney transplant or dialysis for survival. A disturbance in bone metabolism, known as CKD-mineral and bone disorder (CKD-MBD), is a common complication in CKD patients and is found to almost all patients who are on dialysis. CKD-MBD causes altered bone remodeling and bone loss throughout the skeleton and is associated with increased morbidity and mortality^5^. These patients have a high risk of bone fracture due to their low bone mineral density^6^.

A strong association between low bone mass or osteoporosis and fractures has been reported in thalassemia patients^7^. However, it is not clear whether the skeletal changes observed are solely associated with thalassemia or are caused by other complications. The cellular hypoxia in these patients results in increased erythropoietin production, leading to marrow expansion and skeletal deformity^8^. Erythropoietin administration increased cancellous bone loss caused by reduced bone formation and increased bone resorption.

Besides osteoporosis, β-thalassemia major patients have a high risk of developing chronic periodontitis initiated by changes within bacterial biofilm. Periodontitis is an oral infectious disease that affects tissues supporting the tooth and destroys periodontal bone leading to mandibular bone loss. Changes in microbe composition or systemic inflammation also lead to an imbalance between host and oral microbiota. Chronic periodontitis caused by inflammatory reactions to microorganisms in the dental plaque results in the destruction of mandibular alveolar bone. Moreover, periodontal diseases is associated with osteoclast-mediated bone resorption and subsequently tooth loss. It has been reported that thalassemia patients with gingival inflammation have a higher RANKL/OPG ratio in serum and saliva than individuals with a healthy periodontium^9^. Increased levels of IL-6 and IL-8 in gingival crevicular fluid were also observed. Several studies have reported increases in prevalence and severity of periodontal diseases, including gingivitis, and periodontitis in patients with CKD^10,11^.

The aim of the present study was to evaluate the mechanism by which renal insufficiency affected mandibular and femoral bone in thalassemic mice. We used heterozygous β-globin knockout (β^th3/+^, BKO) mice as a model of thalassemia. Mice were 5/6 nephrectomized (Nx) to induce CKD. Our data demonstrated that nephrectomy (Nx) increased bone turnover resulting in decreased cancellous bone volume and cortical thickness in WT. Despite being osteopenic, BKO mice did not exhibit Nx-induced cancellous bone loss and this was possibly due to reduced serum erythropoietin level.

## Results

### BKO mice exhibit microcytic and hypochromic anemia

**Figure 1:**
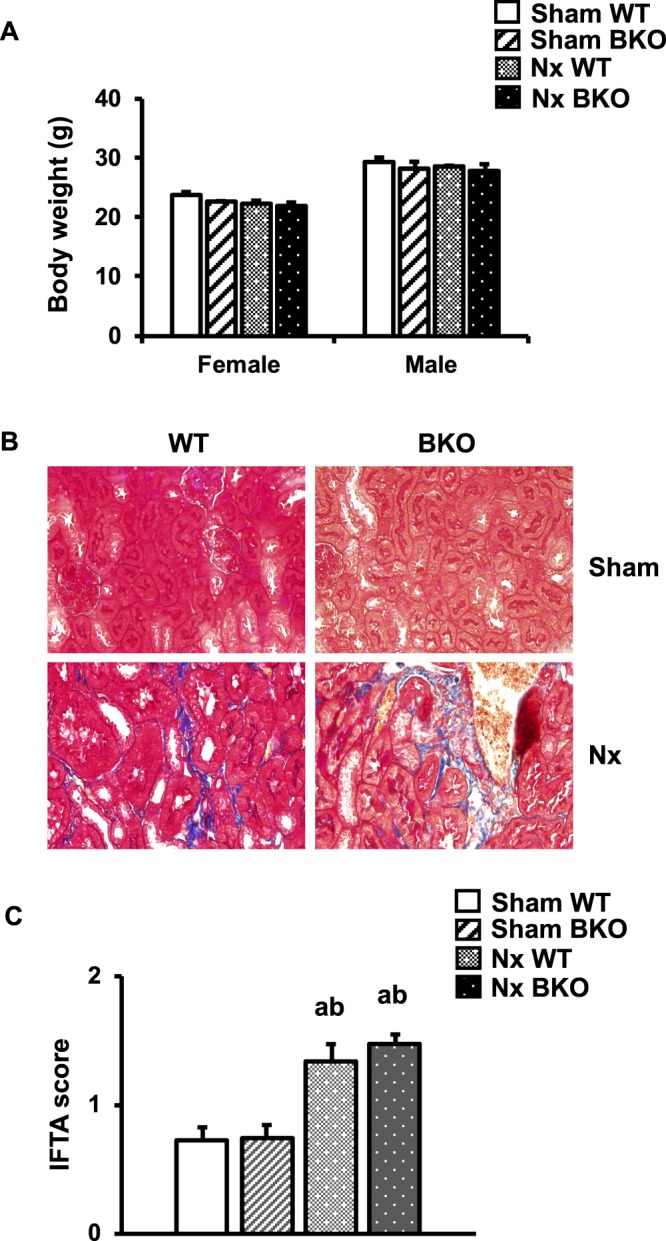
Nx induces renal fibrosis. (**A**) Body weight of BKO and WT controls with Nx. (**B**) Masson’s trichrome staining of the kidney from sham WT, sham BKO, Nx WT and Nx BKO. Blue color indicates fibroblast accumulation. (**C**) IFTA score in the kidneys of sham WT, sham BKO, Nx WT and Nx BKO. Results are mean ± SEM. ^a^*p* < 0.05 versus sham WT, and ^b^*p* < 0.05 versus sham BKO.

Five-month-old female and male sham BKO mice experienced similar weight gain as their sham WT controls (Fig. 1A). Nx did not affect body weight in either WT or BKO mice. The hematological data of the mice are summarized in Supplementary Table S1–2. The hematologic indices of sham BKO mice showed characteristics similar to those of patients with β-thalassemia intermedia. Their red blood cells were microcytic and hypochromic, varied in size and shape (anisocytosis and poikilocytosis) and had uneven hemoglobin distribution showing target cell appearance as a bull’s eye-shaped in the BKO mice (data not shown). In addition, red blood cell (RBC), hemoglobin (Hb), hematocrit (Hct), and mean corpuscular hemoglobin (MCH) values were lower, whereas red cell distribution width-coefficient of variation (RDW-CV) and red cell distribution width-standard deviation (RDW-SD) were higher in sham BKO mice compared to WT controls of both genders. Moreover, mean corpuscular volume (MCV) was decreased in male BKO mice whereas mean corpuscular hemoglobin concentration (MCHC) was reduced in female BKO mice. Nx induced anemia in WT controls and BKO mice. RBC and Hb levels were decreased in female Nx WT and BKO compared to their corresponding controls. In males, Nx decreased RBC, Hb and Hct levels in WT controls and attenuated RBC number in BKO mice. Two-way ANOVA confirmed the effect of BKO and Nx on anemia in both genders. However, Nx did not alter MCV, MCH, RDW-CV or RDW-SD in WT controls and BKO mice. MCHC was decreased in female Nx BKO mice.

### Nx induces renal fibrosis and increases serum urea nitrogen levels in WT and BKO mice

**Figure 2:**
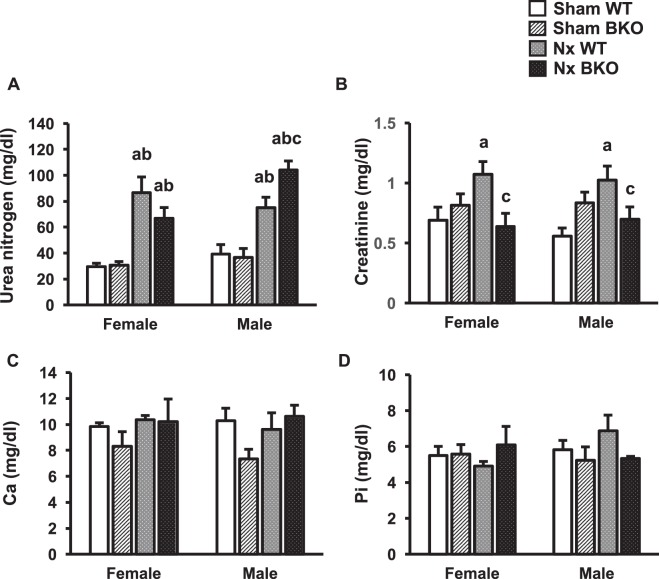
Nx increases serum urea nitrogen levels in both WT and BKO mice. (**A**) serum urea nitrogen. **(B**) serum creatinine. (**C**) serum calcium. (**D**) serum phosphorus. Results are mean ± SEM. ^a^*p* < 0.05 versus sham WT, ^b^*p* < 0.05 versus sham BKO, and ^c^*p* < 0.05 versus Nx WT.

Renal fibrosis is a predictor of an irreversible loss of renal function and progression to end stage renal disease. Sham BKO mice did not have renal fibrosis (Fig. 1B). We determined whether 3 months 5/6 Nx induced renal failure in WT and BKO mice. As shown in Fig. 1B, Nx resulted in comparable tubulointerstitial fibrosis in BKO mice and WT controls, confirming the success of CKD by 5/6 Nx. Thalassemia did not alter the IFTA score, indicating that sham BKO mice had normal kidney function. As expected, Nx increased the IFTA score. However, the score was similar in Nx WT and BKO mice (Fig. 1C). Thalassemia did not affect serum urea nitrogen, creatinine, calcium or phosphorus levels (Fig. 2). In contrast, Nx increased the serum level of urea nitrogen in both WT and BKO mice and the level was much higher in BKO males. Serum creatinine was increased in Nx WT controls but not BKO mice. However, serum calcium and phosphorus levels were not altered in either Nx WT or Nx BKO mice.

### Mandibles of BKO mice exhibit resistance to Nx-induced cancellous bone loss

**Figure 3:**
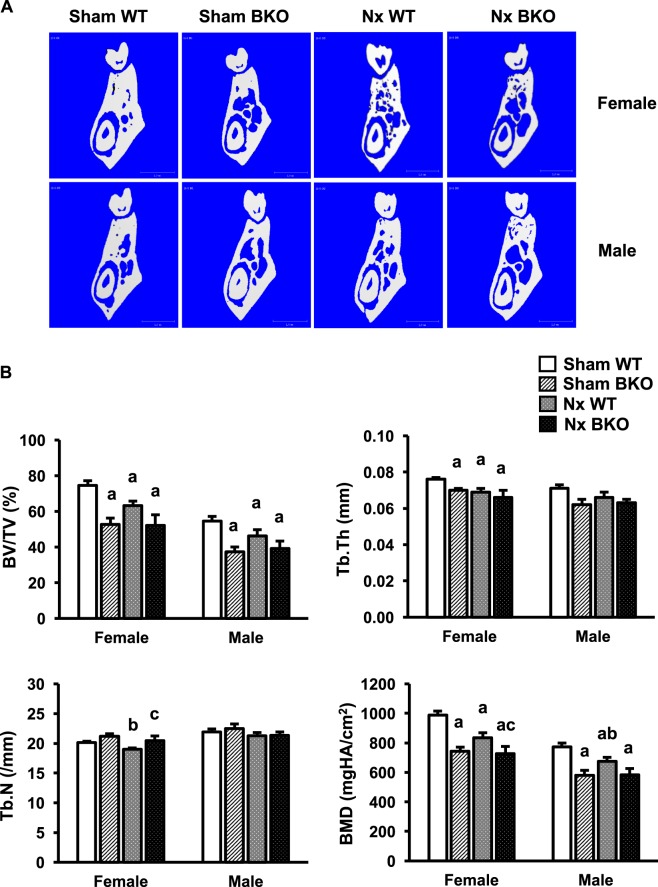
Nx increases mandibular cancellous bone loss in WT but not BKO mice. (**A**) μCT images of the mandibles from sham WT, sham BKO, Nx WT and Nx BKO. (**B**) μCT analysis of cancellous bone of the mandibles. Results are mean ± SEM. ^a^*p* < 0.05 versus sham WT, ^b^*p* < 0.05 versus sham BKO and ^c^*p* < 0.05 versus Nx WT.

**Figure 4:**
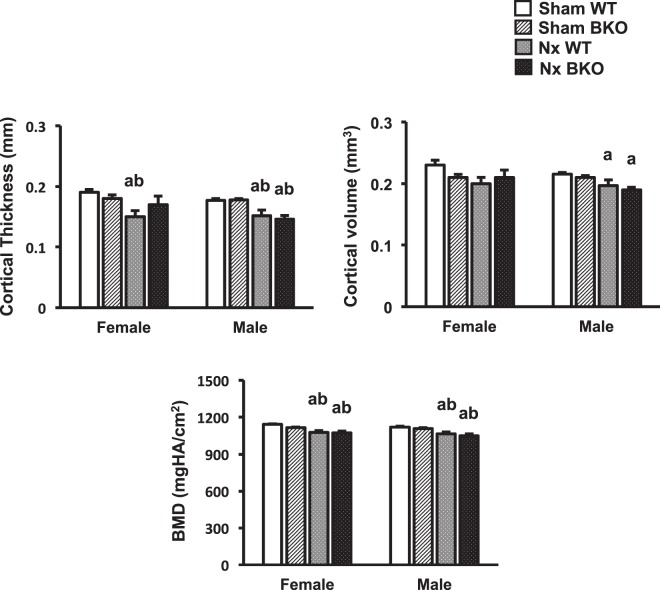
Nx induces mandibular cortical bone loss. μCT analysis of cortical bone of the mandibles. Results are mean ± SEM. ^a^*p* < 0.05 versus sham WT, and ^b^*p* < 0.05 versus sham BKO.

Mandibular cancellous bone volume was decreased in both female and male sham BKO mice (Fig. 3). The decrease in bone volume was due to significant decrease in trabecular thickness in females. Trabecular thickness was also slightly reduced in sham BKO males but the change did not reach statistical significance. Consistent with change in bone volume, bone mineral density was decreased in both male and female sham BKO mice, indicating osteopenic phenotype (Fig. 3). Trabecular number (Fig. 3B) and trabecular separation (data not shown) were not altered in BKO mice of either gender. In control, thalassemia had no effect on cortical bone (Fig. 4). Therefore, thalassemia induced cancellous bone loss in mandibles of both male and female mice. Nx was found to decrease cancellous bone volume and cortical thickness in mandibles of WT females and males (Figs. 3–4). Trabecular thickness was decreased in Nx WT females but not males. In contrast to females, mandibles of male mice exhibited Nx-induced decrease in cortical volume. Bone mineral density of cancellous and cortical bone was lowered in Nx WT controls of both genders when compared to sham WT. Interestingly, BKO mice were resistant to Nx-induced cancellous bone loss. However, cortical bone mineral density was reduced in Nx BKO mice compared to BKO mice in both genders. Cortical thickness was decreased in BKO males following Nx.

### Nx increases osteoblast and osteoclast marker gene expression in WT but not BKO mandibles

**Figure 5:**
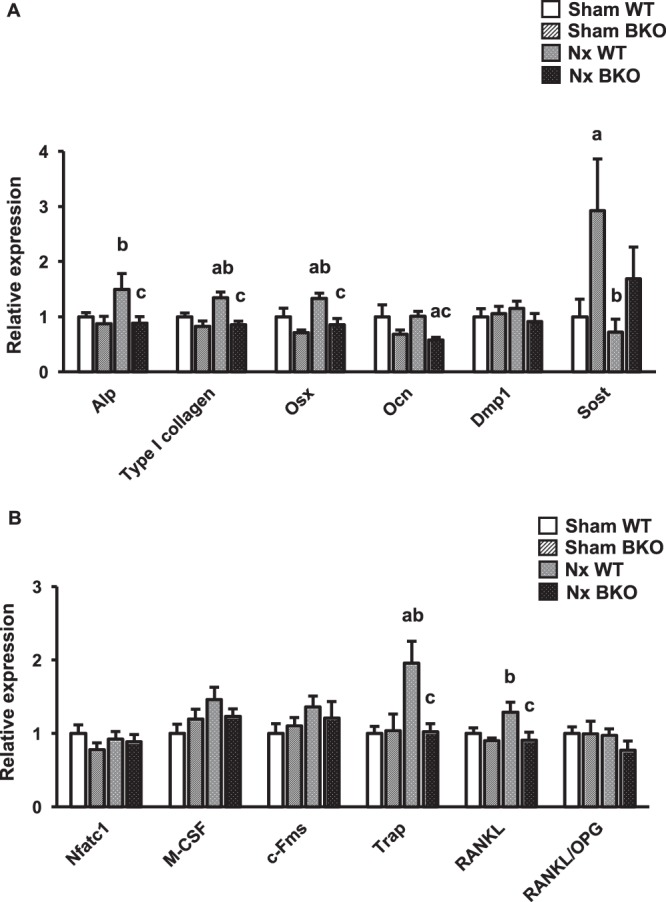
Osteoblast and osteoclast marker gene expression are upregulated in mandibles of Nx WT but not BKO mice. (**A**) qPCR analysis of osteoblast marker genes. (**B**) qPCR analysis of osteoclast marker genes. Results are mean ± SEM. ^a^*p* < 0.05 versus sham WT, ^b^*p* < 0.05 versus sham BKO, and ^c^*p* < 0.05 versus Nx WT.

The differential gene expression profiles of the mandible in Nx WT and Nx BKO males were compared to their corresponding controls (Fig. 5). The qPCR results showed that the expression of osteoblast marker genes, including *Osx* and *Ocn* was slightly decreased in sham BKO mandibles using one-way ANOVA (*p* = 0.08, Fig. 5A). However, two-way ANOVA indicated that thalassemia led to significant decreases in these osteoblast marker gene expression (data not shown). Thalassemia resulted in an approximately 3-fold increase in the *Sost* mRNA level in sham mice. In contrast, *Alp, Type I collagen, Dmp 1* and osteoclast marker gene expression did not change in sham BKO mice. Nx increased the osteoblast markers, *type I collagen* and *Osx* mRNA levels and osteoclast maker genes, *Trap* mRNA level in WT controls (Fig. 5A,B). Other osteoclast markers, including *M-CSF* and *RANKL*, demonstrated slightly increased expression in Nx WT controls. However, *Nfatc1, c-Fms*, and *RANKL/OPG* ratio mRNA levels were not altered by Nx. Nx-induced increase in osteoblast and osteoclast marker gene expression was not observed in BKO mice. These data demonstrated that Nx increased bone turnover with increases in both bone formation and bone resorption in WT but not BKO mice.

### Nx-induced cortical, but not cancellous bone loss in femurs of BKO mice

**Figure 6:**
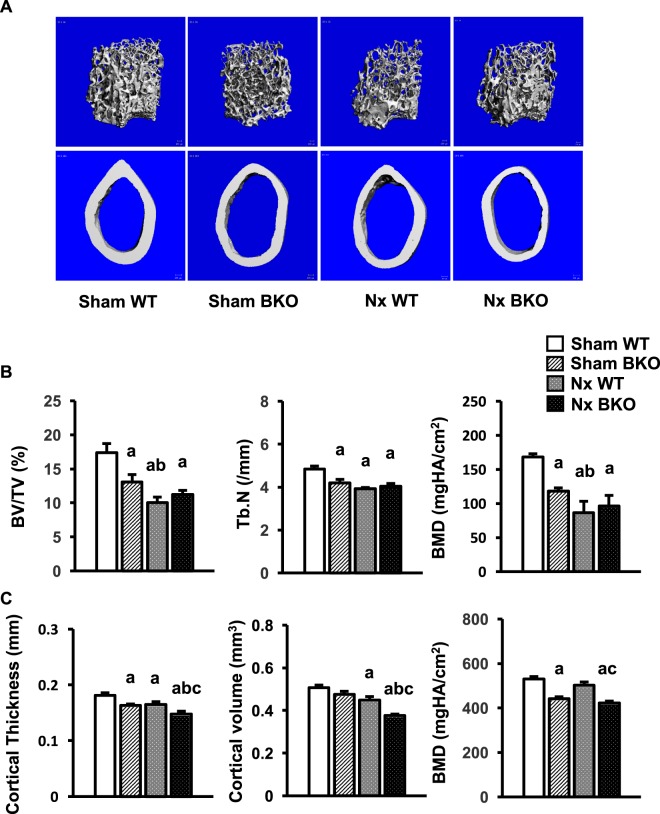
Femoral cancellous bone loss does not worsen in Nx BKO mice. (**A**) μCT images of femoral cancellous (upper) and cortical bone (lower) from sham WT, sham BKO, Nx WT and Nx BKO. (**B**) μCT analysis of cancellous bone of the femurs. (**C**) μCT analysis of cortical bone of the femurs. Results are mean ± SEM. ^a^*p* < 0.05 versus sham WT, ^b^*p* < 0.05 versus sham BKO and ^c^*p* < 0.05 versus Nx WT.

To determine whether the effects of Nx on bone turnover in thalassemic mice were site specific. We examined bone phenotype in femurs of male Nx WT and BKO mice. μCT analysis showed a decrease in cancellous bone volume, trabecular number, cortical thickness, and bone mineral density in both cortical and cancellous bone in sham male BKO mice (Fig. 6A–C). Similar to mandibles, Nx induced cancellous and cortical bone loss in WT. Development of CKD decreased cortical thickness, and cortical bone volume but did not affect cancellous bone in thalassemic mice. Histomorphometric analysis revealed a significant decrease in cancellous bone volume, trabecular thickness, and trabecular number with a concomitant increase in trabecular separation in sham BKO mice (Table 1). The decrease in cancellous bone volume was due to reduced bone formation rate and osteoblast number without any change in osteoclast number. Nx decreased bone volume, trabecular thickness, and trabecular number in WT (Table 1). While mineralizing surface was not altered, mineral apposition rate was increased in Nx WT, leading to increased bone formation rate. Osteoblast and osteoclast number per tissue area were both increased, indicating an increase in bone turnover. All parameters in Nx BKO were not affected when compared to BKO mice.

**Table 1:** Histomorphometric analysis of femurs in BKO mice and WT controls with Nx.

Parameters	Sham		Nx		Two-way ANOVA		
	WT	BKO	WT	BKO	BKO	Nx	Interaction
	(n = 5)	(n = 6)	(n = 6)	(n = 7)			
BV/TV (%)	15.47 ± 1.08	9.22 ± 0.90^a^	10.29 ± 0.68^a^	7.71 ± 0.64^ac^	*p* < 0.05	*p* < 0.05	*p* < 0.05
Tb.Th (μm)	43.38 ± 2.03	34.95 ± 2.12^a^	36.32 ± 2.29^a^	34.70 ± 0.86^a^	*p* < 0.05	NS	NS
Tb.N (/mm)	3.55 ± 0.11	2.63 ± 0.21^a^	2.88 ± 0.22^a^	2.20 ± 0.14^ac^	*p* < 0.05	*p* < 0.05	NS
Tb.Sp (μm)	239 ± 11	358 ± 33^a^	322 ± 27	432 ± 35^ac^	*p* < 0.05	*p* < 0.05	NS
MS/BS (%)	23.64 ± 3.13	15.53 ± 1.61	30.45 ± 1.48^b^	17.58 ± 3.48^c^	*p* < 0.05	NS	NS
MAR (μm/day)	0.97 ± 0.02	0.93 ± 0.04	1.23 ± 0.11^ab^	0.78 ± 0.03^ac^	*p* < 0.05	NS	*p* < 0.05
BFR/TV (%/year)	63.28 ± 7.46	29.64 ± 4.31^a^	80.70 ± 3.36^ab^	25.04 ± 5.41^ac^	*p* < 0.05	NS	*p* < 0.05
N.Ob/T.Ar (/mm^2^)	44.41 ± 4.01	17.02 ± 2.99^a^	65.51 ± 6.64^ab^	20.10 ± 2.22^ac^	*p* < 0.05	*p* < 0.05	*p* < 0.05
N.Oc/T.Ar (/mm^2^)	1.99 ± 0.43	1.67 ± 0.24	4.05 ± 0.75^ab^	1.16 ± 0.38^c^	*p* < 0.05	NS	*p* < 0.05

^a^*p* < 0.05 compared to sham WT, One-way ANOVA followed by Fisher’s PLSD.

^b^*p* < 0.05 compared to sham BKO.

^c^*p* < 0.05 compared to Nx WT.

### Decreased erythropoietin but not FGF23 production in Nx BKO mice

**Figure 7:**
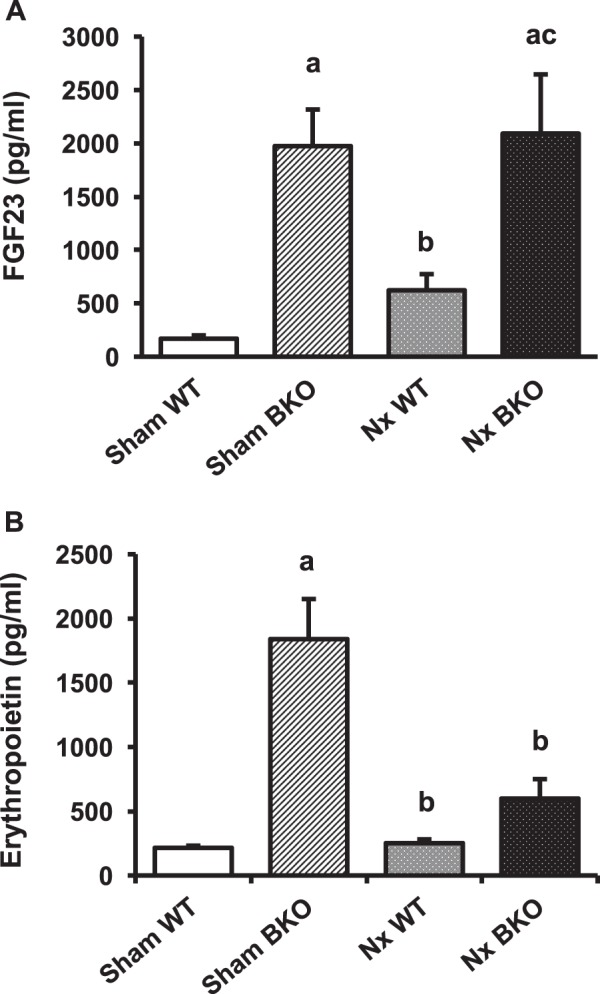
Nx decreases serum erythropoietin but not FGF23 level in BKO mice. (**A**) serum FGF23. (**B**) serum erythropoietin. Results are mean ± SEM. ^a^*p* < 0.05 versus sham WT, ^b^*p* < 0.05 versus sham BKO, and ^c^*p* < 0.05 versus Nx WT.

FGF23, a bone-derived phosphaturic hormone secreted by osteocytes and osteoblasts, regulates mineral homeostasis. Elevated serum FGF23 associated with CKD helped to maintain normal serum phosphate levels. Thalassemia markedly increased serum FGF23 levels as observed in sham BKO mice compared to sham WT controls (Fig. 7A). Nx increased serum FGF23 by 3.4-fold in WT controls but this change was not statistically significant. Nx BKO mice had similar serum FGF23 levels compared to sham BKO mice.

We examine whether Nx affected serum levels of erythropoietin, a hormone released from adult kidney in response to cellular hypoxia to stimulate erythropoiesis in bone marrow. BKO mice had much higher serum erythropoietin level than WT (Fig. 7B). Nx did not alter the serum level of erythropoietin. Interestingly, the serum level of erythropoietin was decreased in BKO mice following 5/6 Nx when compared to sham BKO mice. These data indicated that the observed normal cancellous bone turnover in Nx BKO mice may be due to the lower level of serum erythropoietin.

### Erythropoietin induced cancellous bone loss in WT

**Figure 8:**
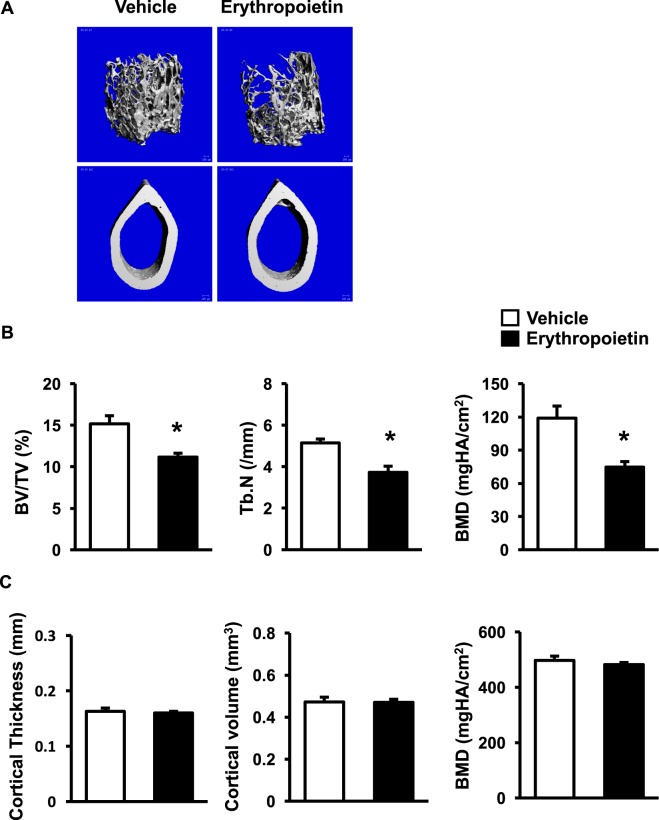
Erythropoietin induces cancellous bone loss. **(A**) μCT images of femoral cancellous (upper) and cortical bone (lower) from WT controls treated with either vehicle or erythropoietin. (**B**) μCT analysis of cancellous bone of the femurs. (**C**) μCT analysis of cortical bone of the femurs. Results are mean ± SEM. *p < 0.05 versus WT.

To examine whether erythropoietin could induce cancellous bone loss, WT were injected with either vehicle or erythropoietin. As expected, RBC (8.54 ± 0.36 vs 12.44 ± 0.54), Hb (12.47 ± 0.55 vs 18.18 ± 0.46), and Hct (39.80 ± 1.53 vs 58.60 ± 1.78) were increased in WT treated with erythropoietin. Moreover, μCT analysis showed that erythropoietin significantly decreased cancellous bone volume, trabecular number and bone mineral density without having effect on cortical bone (Fig. 8A,B). These data confirmed that high serum levels of erythropoietin induced cancellous osteopenia.

## Discussion

Patients with either β-thalassemia or CKD have a high prevalence of low bone mass, fractures, and bone pain. These patients are also at high risk of dental carries, oral infection and periodontal diseases that lead to mandibular alveolar bone loss. Determining the mechanism by which β-thalassemia and CKD induced bone loss is important for understanding the pathology of these diseases on bone metabolism and may lead to new targets for preventing craniofacial deformities and osteoporosis. The present study utilized BKO mice as a mouse model of thalassemia to investigate bone change in thalassemic mice that developed CKD after 5/6 Nx. We showed that BKO mice were anemic and had low cancellous bone volume and mineral density in both mandibles and femurs. Three months after 5/6 Nx, serum urea nitrogen levels were increased and renal fibrosis was observed, confirming the establishment of renal failure. Nx decreased cancellous bone volume and cortical thickness in WT controls. The reduction in cancellous bone volume was associated with high bone turnover in both mandibles and femurs. However, the cancellous bone of BKO mice which was already low at physiological baseline was not affected by Nx. In other words, increased cancellous bone turnover following renal insufficiency was not observed in thalassemic mice. The absence of bone response to renal insufficiency was restricted to cancellous bone since Nx still decreased mandibular cortical bone mineral density in male and female BKO mice and cortical thickness in male BKO mice. Femur cortical thickness and cortical volume were also decreased. The gene expression profiles indicated that Nx-induced increase in osteoblast and osteoclast marker gene expression in mandibles did not occur in thalassemic mice. Histomorphometry showed similar bone formation and bone resorption in femurs of Nx BKO mice and BKO mice. The serum level of erythropoietin was markedly increased in BKO mice but decreased in Nx BKO mice. BKO mice had highly elevated serum FGF23 level but Nx did not have any effect on the serum level of FGF23.

Patients with thalassemia have fewer healthy red blood cells, and less Hb and Hct^12^. MCV and MCH were decreased, indicating the unusually small RBC (microcytosis)^13^. Hypochromic microcytic anemia, smaller RBC and decreased Hb, are commonly caused low MCHC in thalassemia. The elevated RDW is associated with variation in RBC size (anisocytosis) as observed in these patients. Normal RBC should be approximately the same size. Target cells, abnormally shaped RBC with higher cell surface relative to cell volume, are often found in thalassemia, hemoglobinopathy and liver disease. Our BKO mice had hematological characteristics similar to those of thalassemia patients. RBC, Hb, Hct, MCV, MCH and MCHC were decreased whereas RDW-CV and RDW-SD were increased. As expected, Nx induced anemia in both WT and BKO mice. In patients with CKD, anemia begins to develop from an early stage of CKD when patients have 20–50% of normal kidney function, worsen as CKD progresses. It was suggested that CKD associated anemia results from the inadequate renal production of erythropoietin, a hormone that promotes RBC formation in bone marrow^14^.

Patients with β-thalassemia major have a high incidence of various complications. These complications include growth retardation, hypothyroidism, endocrine dysfunction, liver failure, osteoporosis and abnormal renal function^12,15^. Serum urea nitrogen is normally used to assess renal function because it reflects the glomerular filtration rate (GFR). The severity of kidney disease correlates inversely with the GFR. Since BKO mice in this study had normal serum urea nitrogen level, they were assured to have normal renal function. Nx, on the other hand, increased serum levels of urea nitrogen in WT which was consistent with the previous findings^16,17^. Similarly, Nx-induced increase in serum urea nitrogen was observed in BKO mice but the increase was higher than in Nx WT in males. Serum creatinine, a breakdown product of creatine phosphate in muscle and another indicator of impaired renal function was higher in Nx sham WT. However, serum creatinine levels in sham BKO and Nx BKO mice were similar. The inconsistency between serum levels of creatinine and urea nitrogen in Nx BKO mice cannot be explained and needs further investigation. Similar to other reports^16^, our findings showed that Nx did not alter serum levels of calcium or phosphorus.

β-thalassemia major contributes to skeletal deformity, osteopenia and frequent fractures. The pathogenesis of the low bone mass in thalassemia patients includes bone marrow expansion caused by extramedullary hematopoiesis, endocrine dysfunction and iron overload^15^. Some studies reported that the reduced osteoblastic activity was accompanied by a comparable or even greater increase in bone resorption^15^. Previous bone histomorphometry study in BKO mice showed that reduction in cancellous bone volume was due to suppressed bone formation and increased bone resorption^18^. However, it has been reported that suboptimally blood-transfused thalassemia patients with iron overload had delayed bone maturation, focal osteomalacia and decreased bone formation without evidence of increased bone resorption^19,20^. In the present study, sham BKO mice had low cancellous bone volume in the mandibles possibly due to increased *Sost* mRNA expression without change in osteoclast marker gene expression. *Sost*, a negative regulator of bone formation, is highly expressed in osteocytes but low levels of *Sost* are also detected in osteoblasts, chondrocytes and osteoclasts^21,22^. Patients with a *SOST* null mutation have high bone mass, a skeletal phenotype similar to *Sost*^*−/−*^ mice^23,24^. The elevated *Sost* mRNA levels in the present study suggested a decrease in bone formation in sham BKO mice. These data coincide with a clinical report of thalassemia patients having high circulating level of SOST which is associated with low bone mineral density^25^. In addition, BKO mice had decreased bone formation rate and osteoblast number with normal osteoclast number in femurs.

Patients with thalassemia have a high prevalence of dental caries, malocclusion, gingivitis and periodontitis with susceptibility to infection^26,27^. Children who suffer from thalassemia have increased levels of periodontitis and alveolar bone loss. The mandibular alveolar bone loss is a major consequence of chronic periodontitis. The mandibles of patients with thalassemia grow slower and their facial skeletal changes correlate with the severity of their anemia^28^. However, the effects of CKD on mandibular bone remodeling are unresolved. It was reported that CKD resulted in significant increases in cancellous bone volume and trabecular thickness and decreases in cortical bone volume and cortical thickness in the mandibles^29^. Guo Y and coworkers reported a reduction in cortical thickness without any change in cancellous bone^16^. Our results demonstrated that WT with progressive CKD had a mandibular phenotype consistent with clinical periodontal disease. Specifically, Nx WT showed a significantly lower mandibular cancellous bone volume, cortical thickness and bone mineral density in the region of the first molar compared to sham WT. The decrease in bone mineral density was observed in both cancellous and cortical bone. The mRNA expression of *type I collagen*, *Osx* and *Trap* were increased, indicating increased bone turnover in Nx WT controls. Histomorphometric analysis also showed increases in bone formation and bone resorption in femurs of Nx WT. In contrast, BKO mice were resistant to Nx-induced cancellous bone loss.

FGF23 is associated with erythropoiesis and bone mineralization. Serum level of FGF23 was markedly increased in BKO mice together with high serum erythropoietin level. These data were consistent with the report of transgenic erythropoietin-overexpressing mice having elevated circulating FGF23^30^. Nx slightly increased the serum levels of FGF23 in WT but not BKO mice. Although FGF23 has direct effects on bone turnover following CKD development, our data suggest that FGF23 may not be the key factor that regulates bone remodeling in BKO mice after Nx.

BKO mice had high circulating level of erythropoietin similar to thalassemia patients^31^. Chronic anemia in thalassemia markedly stimulates the production of erythropoietin up to 20–30 times normal level with consequent massive medullar cell proliferation. However, upregulated erythropoiesis is ineffective because the high numbers of erythroid precursors fail to develop into mature red blood cells. Nevertheless, the increased marrow erythropoiesis is one of the major determinants of reduced bone mass in thalassemia patients^32^. Endogenous erythropoietin is known to be essential for bone marrow stromal cell differentiation into osteoblasts and bone microenvironment. However, the effects of erythropoietin on bone homeostasis are controversial. There have been a number of investigations on erythropoietin and bone turnover. Erythropoietin treatment increased bone formation leading to increased bone mass in newborn and growing mice^33^, whereas it induced bone loss in adult mice^34^. Our present data confirmed that high level of erythropoietin induced cancellous bone loss in WT. Transgenic mice overexpressing human erythropoietin also exhibited low bone mass, decreased bone formation and increased bone resorption^35^. Erythropoietin directly stimulated osteoclastogenesis via activation of Jak2 and PI3K. On the other hand, the effects of erythropoietin on osteoblasts were indirect since erythropoietin administration did not inhibit osteoblast differentiation *in vitro*. It is possible that lower level of erythropoietin in Nx BKO mice when compared to intact BKO mice might be responsible for the protection against cancellous bone loss in these mice. Furthermore, abnormal proliferation of bone marrow cells independent of hematopoietic lineage such as in hemolytic anemias, chronic myeloproliferative disorders and cancers was reported to be associated with bone loss^36^. Thus, in addition to direct effect of erythropoietin on osteoblast and osteoclast, lower level of erythropoietin in Nx BKO mice may result in less erythropoiesis associated marrow expansion and subsequently less cancellous bone loss in Nx BKO mice. Besides erythropoietin, uremic toxins, including indoxyl sulfate and p-cresol sulfate are major causes of bone abnormalities in patients with CKD^37^. The resistance to cancellous osteopenia in BKO mice after Nx may be due to different uremic factors produced in thalassemia. However, further studies will be required to determine the detailed mechanisms by which uremic toxins affect bone turnover in thalassemic mice. For clinical translation, physicians should evaluate BMD and serum levels of erythropoietin in thalassemia patients with CKD before initiating treatment since the reduced erythropoietin might already protect against bone loss in these patients.

In conclusion, BKO mice exhibited low cancellous bone volume and mineral density. In WT, Nx increased bone turnover, leading to cancellous and cortical bone loss. However, Nx-induced cancellous bone loss was not observed in BKO mice and this was likely to be due to lower circulating level of erythropoietin after Nx.

## Materials and Methods

### Animals

BKO mice^38^ were obtained from the Thalassemia Research Center, Institute of Molecular Biosciences, Mahidol University. All animal procedures were approved by the Institutional Animal Care and Use Committee at Faculty of Medicine, Chulalongkorn University. Mice were housed at the Faculty of Medicine, Chulalongkorn University and had free access to water and standard rodent chow. They were maintained in accordance with the Guide for the Care and Use of Laboratory Animals (eighth edition), National Research Council.

Homozygous BKO mice were embryonically lethal. Female and male BKO mice were crossed to generate BKO mice and their WT controls. The heterozygotes and WT controls were genotyped by blood smear. Seven-week-old females and males were divided into 4 groups; sham WT, sham BKO, Nx WT and Nx BKO. For the CKD animal model, 5/6 Nx was performed in two stages under isoflurane anesthesia. At week -1, a left flank incision was performed to resect two-thirds of the left kidney (upper and lower poles). Bleeding was controlled by microfibrillar collagen hemostasis (Avitene, Davol, Cranston, RI). One week later (week 0), the entire right kidney was removed via a right flank incision. For the sham-operated mice, the flank incision was performed without kidney removal and the incision was closed. Mice were subcutaneously injected with 20 mg/kg calcein and 40 mg/kg tetracycline at 8 and 2 days prior to animal necropsy. Three months after surgery, mice were anesthetized with isoflurane. Blood samples were collected in a tube containing EDTA for complete blood count analysis at the Faculty of Veterinary Science, Chulalongkorn University. The remaining blood samples were centrifuged and the serum was kept at -80 °C for determination of urea nitrogen, creatinine, calcium, phosphorus, FGF23 and erythropoietin levels. The mandibles were removed and the left mandibles were fixed in 10% neutral buffered formalin for microcomputed tomography (μCT) analysis. The right mandibles were frozen in liquid nitrogen and kept at −80 °C for RNA isolation and qPCR analysis. Left femurs were fixed in 70% alcohol for μCT and histomorphometric analysis. The left kidney was removed for histological studies.

For erythropoietin experiment, 7-week-old WT males were subcutaneously injected with 8 doses of either vehicle (PBS) or 180 IU erythropoietin (Roche, Basel, Switzerland) 3 times a week over 2.5 weeks^35^. At the end of the experiment, blood samples were collected for complete blood count analysis. Left femurs were removed and fixed in 70% alcohol for μCT analysis.

### μCT analysis

High resolution images of a buccal-lingual cross slice of the first mandibular molars and femurs were acquired using a desktop μCT35, (Scanco Medical, Basserdorf, Switzerland) in accordance with recommended guidelines^39^. The cancellous and cortical bone microarchitecture was determined using a 7 μm isotropic voxel size, 50 kVp, and 144 μA. Mandibular bone scans were subjected to Gaussian filtration and segmentation using a fixed threshold of 330. Femur was evaluated at a threshold of 220 and 350 for cancellous and cortical bone, respectively. Bone volume fraction (BV/TV, %), trabecular number (Tb.N, /mm), trabecular thickness (Tb.Th, mm), trabecular separation (Tb.Sp, mm), cortical volume (mm^3^), cortical thickness (mm) and bone mineral density (mgHA/cm^2^) were analyzed.

### Real-time quantitative PCR (qPCR)

Total RNA was isolated from the right mandibles using a monophasic solution of guanidine isothiocyanate and phenol as indicated in the manufacturer’s protocol (Trizol Reagent; Invitrogen, Carlsbad, CA, USA). The RNA was cleaned up using an RNeasy Mini kit (Qiagen, Germantown, MD, USA) and the RNA yields were determined spectrophotometrically at 260 nm. The cDNA was synthesized from 1 μg of total RNA with SuperScript VILO cDNA synthesis kit (Invitrogen, Carlsbad, CA, USA) for reverse transcription. The qPCR was performed at 60 °C for 40 cycles using CFX96^TM^ Optics Module (Bio-Rad). The expression levels were normalized to GAPDH expression. Supplementary Table S3 shows the oligonucleotide primers for qPCR analysis.

### Bone histomorphometry

Femurs were dehydrated in 70, 90 and 100% acetone, infiltrated and embedded in methyl methacrylate. Undecalcified sections were cut at a thickness of 5 μm using a motorized microtome (RM2255, Leica, Germany). The unstained sections were used to analyze fluorescent labeling for dynamic measurements and the consecutive sections were stained with toluidine blue to quantify static measurements as previously described^40^. Cancellous bone was evaluated in the distal metaphysis of femurs at 400 μm below the growth plate using OsteoMeasure system (Osteometrics Inc., Decatur, GA), and all parameters were analyzed following the standardized nomenclature^41^. A sampling site had area of approximately 2.2 mm^2^.

### Renal histology

The left kidney was dehydrated and embedded in paraffin. The kidney interstitial fibrosis and tubular atrophy (IFTA) score was estimated at 20X magnification on 5 µm thick sections stained with Masson’s Trichrome. Ten fields were randomly scored using a semi-quantitative ordinal scale: 0, damage involving <5%; 1, damage involving 5–10%; 2, area of damage 11–25%; 3, area of damage 26–50%; and 4, >50% of the area being affected over the entire kidney section^42^.

### Serum chemistry

Serum urea nitrogen, creatinine, calcium, and phosphorus levels were assessed according to the manufacturer’s instructions (Standbio Laboratory, Boerne, TX). Serum level of erythropoietin was measured by ELISA kit (R&D systems, Minneapolis, MN) as per manufacturer’s protocol. Serum FGF23 was measured using the mouse/rat FGF-23 (C-Term) ELISA kit (Quidel, San Diego, CA) which detected both intact and c-terminal fragments of FGF23.

### Statistical analysis

All data were expressed as mean ± SEM. The results were analyzed for significant differences using one-way ANOVA followed by Fisher’s protected least significant difference test. The effects of genotype and Nx and interactions between genotype and Nx were determined using two-way ANOVA. Statistical significance was defined as *p* < 0.05.

## Supplementary information

Supplementary Table.

## Data Availability

All data are available from the corresponding author upon request.

## References

[1] Rachmilewitz EA, Giardina PJ (2011). How I treat thalassemia. Blood.

[2] Borgna-Pignatti C (2006). Cardiac morbidity and mortality in deferoxamine- or deferiprone-treated patients with thalassemia major. Blood.

[3] Berdoukas V (2009). The efficacy of iron chelator regimes in reducing cardiac and hepatic iron in patients with thalassaemia major: a clinical observational study. J. Cardiovasc. Magn. Reson..

[4] Ziyadeh FN (2012). Glomerular hyperfiltration and proteinuria in transfusion-independent patients with beta-thalassemia intermedia. Nephron Clin. Pract..

[5] Bover J, Cozzolino M (2011). Mineral and bone disorders in chronic kidney disease and end-stage renal disease patients: new insights into vitamin D receptor activation. Kidney Int Suppl (2011).

[6] Ott SM (2013). Therapy for patients with CKD and low bone mineral density. Nat Rev Nephrol.

[7] Vogiatzi MG (2009). Bone disease in thalassemia: a frequent and still unresolved problem. J. Bone Miner. Res..

[8] Wong P, Fuller PJ, Gillespie MT, Milat F (2016). Bone Disease in Thalassemia: A Molecular and Clinical Overview. Endocr. Rev..

[9] Akcali A (2015). The Association Between Thalassemia Major and Periodontal Health. J. Periodontol..

[10] Gavalda C (1999). Renal hemodialysis patients: oral, salivary, dental and periodontal findings in 105 adult cases. Oral Dis..

[11] Davidovich E, Schwarz Z, Davidovitch M, Eidelman E, Bimstein E (2005). Oral findings and periodontal status in children, adolescents and young adults suffering from renal failure. J. Clin. Periodontol..

[12] Karim MF, Ismail M, Hasan AM, Shekhar HU (2016). Hematological and biochemical status of Beta-thalassemia major patients in Bangladesh: A comparative analysis. Int J Hematol Oncol Stem Cell Res.

[13] Akhavan-Niaki H (2012). Hematologic features of alpha thalassemia carriers. Int J Mol Cell Med.

[14] Cases A (2018). Anemia of chronic kidney disease: Protocol of study, management and referral to Nephrology. Nefrologia.

[15] Voskaridou E, Terpos E (2004). New insights into the pathophysiology and management of osteoporosis in patients with beta thalassaemia. Br. J. Haematol..

[16] Guo Y (2016). Estrogen Deficiency Leads to Further Bone Loss in the Mandible of CKD Mice. PLoS One.

[17] Sun N (2015). FGF23 neutralization improves bone quality and osseointegration of titanium implants in chronic kidney disease mice. Sci. Rep..

[18] Thongchote K, Svasti S, Teerapornpuntakit J, Krishnamra N, Charoenphandhu N (2014). Running exercise alleviates trabecular bone loss and osteopenia in hemizygous beta-globin knockout thalassemic mice. Am. J. Physiol. Endocrinol. Metab..

[19] Mahachoklertwattana P (2003). Bone histomorphometry in children and adolescents with beta-thalassemia disease: iron-associated focal osteomalacia. J. Clin. Endocrinol. Metab..

[20] Mahachoklertwattana P (2003). Bone mineral density, biochemical and hormonal profiles in suboptimally treated children and adolescents with beta-thalassaemia disease. Clin. Endocrinol. (Oxf.).

[21] Sebastian A, Loots GG (2017). Transcriptional control of Sost in bone. Bone.

[22] Ota K (2013). Sclerostin is expressed in osteoclasts from aged mice and reduces osteoclast-mediated stimulation of mineralization. J. Cell. Biochem..

[23] Balemans W (2001). Increased bone density in sclerosteosis is due to the deficiency of a novel secreted protein (SOST). Hum. Mol. Genet..

[24] Collette NM (2012). Targeted deletion of Sost distal enhancer increases bone formation and bone mass. Proc. Natl. Acad. Sci. USA.

[25] Voskaridou E (2012). High circulating sclerostin is present in patients with thalassemia-associated osteoporosis and correlates with bone mineral density. Horm. Metab. Res..

[26] Hattab FN (2012). Periodontal condition and orofacial changes in patients with thalassemia major: a clinical and radiographic overview. J. Clin. Pediatr. Dent..

[27] Al-Wahadni AM, Taani DQ, Al-Omari MO (2002). Dental diseases in subjects with beta-thalassemia major. Community Dent. Oral Epidemiol..

[28] Drew SJ, Sachs SA (1997). Management of the thalassemia-induced skeletal facial deformity: case reports and review of the literature. J. Oral Maxillofac. Surg..

[29] Lee MM (2010). Characterization of mandibular bone in a mouse model of chronic kidney disease. J. Periodontol..

[30] Hanudel MR (2019). Effects of erythropoietin on fibroblast growth factor 23 in mice and humans. Nephrol. Dial. Transplant..

[31] Nisli G, Kavakli K, Aydinok Y, Oztop S, Cetingul N (1997). Serum erythropoietin levels in patients with beta thalassemia major and intermedia. Pediatr. Hematol. Oncol..

[32] Mahachoklertwattana P (2006). Association between bone mineral density and erythropoiesis in Thai children and adolescents with thalassemia syndromes. J. Bone Miner. Metab..

[33] Shiozawa Y (2010). Erythropoietin couples hematopoiesis with bone formation. PLoS One.

[34] Singbrant S (2011). Erythropoietin couples erythropoiesis, B-lymphopoiesis, and bone homeostasis within the bone marrow microenvironment. Blood.

[35] Hiram-Bab S (2015). Erythropoietin directly stimulates osteoclast precursors and induces bone loss. FASEB J..

[36] Steer K, Stavnichuk M, Morris M, Komarova SV (2017). Bone Health in Patients With Hematopoietic Disorders of Bone Marrow Origin: Systematic Review and Meta- Analysis. J. Bone Miner. Res..

[37] Liu, W. C., Tomino, Y. & Lu, K. C. Impacts of Indoxyl Sulfate and p-Cresol Sulfate on Chronic Kidney Disease and Mitigating Effects of AST-120. *Toxins* (Basel) **10** (2018). 10.3390/toxins10090367 PMC6162782 30208594

[38] Yang B (1995). A mouse model for beta 0-thalassemia. Proc. Natl. Acad. Sci. USA.

[39] Bouxsein ML (2010). Guidelines for assessment of bone microstructure in rodents using micro-computed tomography. J. Bone Miner. Res..

[40] Lotinun S (2013). Osteoclast-specific cathepsin K deletion stimulates S1P-dependent bone formation. J. Clin. Invest..

[41] Dempster DW (2013). Standardized nomenclature, symbols, and units for bone histomorphometry: a 2012 update of the report of the ASBMR Histomorphometry Nomenclature Committee. J. Bone Miner. Res..

[42] Leelahavanichkul A (2010). Angiotensin II overcomes strain-dependent resistance of rapid CKD progression in a new remnant kidney mouse model. Kidney Int..

