# Supplementary material for: Nephrectomy Does not Exacerbate Cancellous Bone loss in Thalassemic Mice
Source: Sci Rep. 2020 May 8;10:7786. doi: 10.1038/s41598-020-64681-2 (PMC7210954; doi:10.1038/s41598-020-64681-2)
Supplement: Supplementary file 1 — Supplementary Table. [file 41598_2020_64681_MOESM1_ESM.pdf]

Nephrectomy Does not Exacerbate Cancellous Bone loss in Thalassemic Mice

Sutada Lotinun[1,2], Korakot Atjanasuppat[2], Jutatip Limsuvech[2], Asada Leelahavanichkul[3], Saovaros Svasti[4], Nateetip Krishnamra[5]

[1]Department of Physiology, Faculty of Dentistry, Chulalongkorn University, Bangkok, Thailand
[2]Skeletal Disorders Research Unit, Faculty of Dentistry, Chulalongkorn University, Bangkok, Thailand
[3]Department of Microbiology, Faculty of Medicine, Chulalongkorn University, Bangkok, Thailand
[4]Thalassemia Research Center, Institute of Molecular Biosciences, and Department of Biochemistry, Faculty of Science, Mahidol University, Bangkok, Thailand
[5]Department of Physiology, Faculty of Science, Mahidol University, Bangkok, Thailand.

Address correspondence to:

Sutada Lotinun, Ph.D.
Department of Physiology
Faculty of Dentistry
Chulalongkorn University
Bangkok, Thailand
Tel: +662 218-8694
Fax: +662 218-8691
Email: sutada.l@chula.ac.th

Table S1 Hematological data of female BKO and WT controls with Nx

Parameters	Sham		Nx		Two-way ANOVA		
	WT (n=5)	BKO (n=5)	WT (n=5)	BKO (n=3)	BKO	Nx	Interaction
RBC ($x10^6/\mu l$)	8.85±0.34	7.01±0.26[a]	6.83±0.47[a]	5.24±0.43[abc]	$p < 0.05$	$p < 0.05$	NS
Hb (g/dl)	13.46±0.55	7.98±0.14[a]	9.88±0.71[ab]	6.00±0.15[abc]	$p < 0.05$	$p < 0.05$	NS
Hct (%)	44.62±1.65	30.04±1.17[a]	32.70±2.30[a]	24.83±1.34[ac]	$p < 0.05$	$p < 0.05$	NS
MCV (fl)	50.44±0.47	42.90±1.67	47.84±0.39	48.20±5.38	NS	NS	NS
MCH (pg)	15.20±0.10	11.42±0.22[a]	14.44±0.17[b]	11.57±0.77[ac]	$p < 0.05$	NS	NS
MCHC (g/dl)	30.12±0.29	26.70±0.98[a]	30.14±0.19[b]	24.27±1.13[abc]	$p < 0.05$	NS	NS
RDW-CV (%)	15.80±0.60	46.56±3.00[a]	17.32±0.27[b]	44.00±1.22[ac]	$p < 0.05$	NS	NS
RDW-SD (fl)	34.18±1.56	85.60±9.58[a]	35.78±0.73[b]	93.63±13.75[ac]	$p < 0.05$	NS	NS

[a]$p<0.05$ compared to sham WT, One-way ANOVA followed by Fisher's PLSD
[b]$p<0.05$ compared to sham BKO
[c]$p<0.05$ compared to Nx WT

Table S2 Hematological data of male BKO and WT controls with Nx

Parameters	Sham		Nx		Two-way ANOVA		
	WT (n=6)	BKO (n=5)	WT (n=5)	BKO (n=3)	BKO	Nx	Interaction
RBC ($x10^6/\mu l$)	8.36±0.15	6.81±0.37[a]	6.96±0.48[a]	5.14±0.47[abc]	$p < 0.05$	$p < 0.05$	NS
Hb (g/dl)	12.75±0.23	7.22±0.23[a]	10.14±0.72[ab]	6.27±1.27[ac]	$p < 0.05$	$p < 0.05$	NS
Hct (%)	42.62±0.62	24.72±1.18[a]	33.94±2.24[ab]	21.17±4.30[ac]	$p < 0.05$	$p < 0.05$	NS
MCV (fl)	51.05±0.51	36.38±1.07[a]	48.82±0.41[b]	40.50±4.69[ac]	$p < 0.05$	NS	NS
MCH (pg)	15.28±0.08	10.66±0.24[a]	14.54±0.09[b]	11.97±1.38[ac]	$p < 0.05$	NS	$p < 0.05$
MCHC (g/dl)	29.95±0.35	29.36±0.82	29.80±0.27	29.57±0.03	NS	NS	NS
RDW-CV (%)	16.13±0.54	34.80±2.94[a]	17.02±0.54[b]	30.07±7.37[ac]	$p < 0.05$	NS	NS
RDW-SD (fl)	35.57±1.55	53.04±6.20[a]	35.98±1.26[b]	49.03±8.52	$p < 0.05$	NS	NS

[a]$p<0.05$ compared to sham WT, One-way ANOVA followed by Fisher's PLSD
[b]$p<0.05$ compared to sham BKO
[c]$p<0.05$ compared to Nx WT

Table S3 Oligonucleotide primers for qPCR analysis

Gene	Forward primer	Reverse primer
Alp	CTTGACTGTGGTTACTGCTGATCA	GTATCCACCGAATGTGAAAACGT
Type I collagen	CCCAAGGAAAAGAAGCACGTC	ACATTAGGCGCAGGAAGGTCA
Osterix	CCCTTCTCAAGCACCAATGG	AAGGGTGGGTAGTCATTTGCATA
Osteocalcin	GCTGCCCTAAAGCCAAACTCT	AGAGGACAGGGAGGATCAAGTTC
Dmp1	ATGACTGTCAGGACGGCTAC	AGTTATAGTGAACTCTCTAC
Sost	ATCATTTCCAGACACCTCTTAC	ATGTGCTTCTGTTACAAACGCTC
Nfatc1	AGGCTGGTCTTCCGAGTTCA	ACCGCTGGGAACACTCGAT
M-CSF	ACCTGTTTCCCAAGAAGAGAGCCT	AGCTGTCAACACAAGCAGCCAAAG
c-Fms	TGGCATCTGGCTTAAGGTGAA	GAATCCGCACCAGCTTGCTA
Trap	GATCCCTCTGTGCGACATCA	CCAGGGAGTCCTCAGATCCA
OPG	AAGAGCAAACCTTCCAGCTGC	CACGCTGCTTTCACAGAGGTC
RANKL	CAAGCTCCGAGCTGGTGAAG	CCTGAACTTTGAAAGCCCCA
GAPDH	TGCACCACCAACTGCTTAG	GGATGCAGGGATGATGTTC
